# Cardiopulmonary Exercise Testing in People With Dyspnea With a Recent Acute Pulmonary Embolism

**DOI:** 10.1016/j.chpulm.2025.100164

**Published:** 2025-03-18

**Authors:** Dieuwke Luijten, Josien van Es, Jannie J. Abbink, Stefano Barco, Johanna M.W. van den Berg, Waleed Ghanima, Menno V. Huisman, Coen van Kan, Bas Langeveld, Ivo van der Lee, Rosa Mali, Thijs E. van Mens, Timothy A. Morris, Maria Overbeek, Mart van der Plas, Martijn A. Spruit, Frederikus A. Klok, Anton Vonk Noordegraaf, Maarten K. Ninaber

**Affiliations:** a Department of Medicine - Thrombosis and Hemostasis, Leiden University Medical Center, Leiden, The Netherlands; b Department of Pulmonology, Amsterdam UMC, VU University Medical Centre, Amsterdam, The Netherlands; c Department of Pulmonology, OLVG, Amsterdam, The Netherlands; d Basalt Rehabilitation Center, Leiden, The Netherlands; e Department of Angiology, University Hospital of Zurich, Zurich, Switzerland; f Department of Pulmonology, Haga Teaching Hospital, The Hague, The Netherlands; g Departments of Oncology, Medicine and Research, Ostfold Hospital Trust, Kalnes, Norway; h Department of Pulmonology, Deventer ziekenhuis, Deventer, The Netherlands; i Department of Pulmonology, Spaarne gasthuis, Haarlem, The Netherlands; j Department of Pulmonology, Leiden University Medical Center, Leiden, The Netherlands; k Division of Pulmonary and Critical Care Medicine, University of California at San Diego, La Jolla, CA; l Department of Pulmonology, Haaglanden Medisch Centrum, The Hague, The Netherlands; m Department of Research & Development, Ciro, Horn, The Netherlands; n Department of Respiratory Medicine, Maastricht University Medical Centre, NUTRIM Institute of Nutrition and Translational Research in Metabolism, Faculty of Health, Medicine and Life Sciences, Maastricht, The Netherlands

**Keywords:** exercise test, exercise tolerance, pulmonary embolism

## Abstract

**Background:**

Cardiopulmonary exercise testing (CPET) may provide a helpful tool to assess underlying causes of dyspnea in patients with acute pulmonary embolism (PE). However, the response to exercise in the first weeks after diagnosis of an acute PE is currently unknown.

**Research Question:**

What are the cardiopulmonary responses to and safety of performing strenuous exercise within 2 to 4 weeks postacute PE?

**Study Design and Methods:**

A total of 100 patients with acute PE, without major comorbidities, experiencing dyspnea (Medical Research Council dyspnea scale ≥ 2) and functional limitations (Post-Venous Thromboembolism Functional Status Scale grade ≥ 2) 1 to 2 weeks after PE diagnosis, underwent CPET within 2 to 4 weeks after diagnosis. We evaluated the frequency of peak oxygen consumption < 80% predicted, a peak oxygen pulse < 80% predicted or oxygen pulse_AT_/oxygen pulse_rest_ < 2.6, and a ventilatory equivalent for carbon dioxide ≥ 34 at anaerobic threshold or dead space to tidal volume ratio > 30% at peak, and their association with markers of PE severity at diagnosis.

**Results:**

There were no adverse events related to the procedure. CPET disclosed peak oxygen consumption < 80% predicted in 23% of patients, oxygen pulse < 80% predicted or oxygen pulse_AT_/oxygen pulse_rest_ < 2.6 in 75%, and ventilatory equivalent for carbon dioxide at anaerobic threshold ≥ 34 or peak dead space to tidal volume ratio > 30% in 49%. In 1 of 7 patients, none of the previously reported signs were present (14%). Intermediate-high risk PE and central PE were associated with increased incidence of these abnormalities.

**Interpretation:**

There were no complications when performing strenuous exercise in the first weeks after a PE diagnosis in this study. Despite dyspnea, 1 of 7 patients had adequate cardiopulmonary reserve, suggesting that post-PE symptoms are multifactorial. Intermediate-high risk and central PE were associated with higher incidences of abnormal CPET outcomes.

**Clinical Trial Registration:**

Dutch Trial Register; No.: NTR NL9615; URL: https://onderzoekmetmensen.nl/en/trial/54292

**Take-Home Points**

**Study Question:** What are the cardiopulmonary responses to and safety of performing strenuous exercise within 2 to 4 weeks postacute pulmonary embolism (PE) and what is the relationship with the severity of the index PE event?

**Results:** There were no adverse events when performing a strenuous exercise test within 2 to 4 weeks after PE diagnosis in this study. Despite dyspnea, 1 of 7 patients had adequate cardiopulmonary response to exercise.

**Interpretation:** Our results indicate that performing strenuous exercise shortly after PE is safe. Post-PE symptoms seem multifactorial because not all post-PE dyspnea and/or functional limitation can be explained by an abnormal cardiopulmonary limitation when performing exercise.

Acute pulmonary embolism (PE) causes ventilation-perfusion mismatch contributing to hypoxemia and increased pulmonary artery pressure, potentially resulting in right ventricular (RV) failure and obstructive shock.^1^, ^2^, ^3^ Anticoagulant treatment should lead to progressive thrombus resolution and hemodynamic improvement. However, up to 50% of acute PE survivors report persistent symptoms after 3 months, indicating post-PE syndrome.^4^, ^5^, ^6^, ^7^, ^8^, ^9^, ^10^ Three possible PE-related reasons may explain persistent symptoms during follow-up: (1) incomplete thrombus resolution causing chronic thromboembolic pulmonary hypertension or chronic thromboembolic pulmonary disease without pulmonary hypertension in rest (ie, thromboembolism with other physiological defects), (2) incomplete recovery of the right ventricle without residual pulmonary vascular obstruction, and/or (3) post-PE functional impairment without physiological cardiopulmonary defects during exercise (patients without chronic thromboembolic pulmonary disease [eg, exercise intolerance due to deconditioning]).^11^^,^^12^ Preexisting comorbidities and functional limitation may also play an additive role.^8^^,^^13^

Identification of factors limiting exercise tolerance in a particular patient might enhance outcomes.^11^ Cardiopulmonary exercise testing (CPET) is a useful diagnostic test for this purpose.^14^ A study showed a 50% prevalence of abnormal cardiopulmonary limitations 3 to 12 months after PE.^15^ However, the response to exercise and subsequent limitations in the first weeks after acute PE are unknown. Early exercise training programs may help prevent post-PE functional impairment, but further research is needed to understand their safety and the underlying pathophysiology of exercising soon after PE diagnosis.^11^^,^^16^

In this study, we aimed to provide descriptive insight into CPET shortly after PE by investigating the safety of performing CPET shortly after diagnosis, provide descriptive insight into the cardiopulmonary response to exercise within the 2 to 4 weeks after acute PE, and correlate CPET outcomes with markers of PE severity.

## Study Design and Methods

### Study Population and Procedures

This is a preplanned subanalysis of the ongoing PE@HOME study. The PE@HOME study is a prospective, multicenter, randomized controlled trial performed in The Netherlands aimed to evaluate the effect of an exercise training program on exercise tolerance and risk reduction of post-PE syndrome in patients with acute PE. Patients were eligible if they were ≥ 18 years of age, had a CT pulmonary angiography (CTPA)-confirmed PE, and reported incomplete recovery at 1 to 2 weeks after acute PE (ie, persistent dyspnea assessed by a Medical Research Counsel score ≥ 2 and persistent function limitations assessed by a Post-Venous Thromboembolism Functional Status Scale grade ≥ 2).^17^^,^^18^ Exclusion criteria were the following: a life expectancy < 6 months, chronic dyspnea from a known or suspected cardiopulmonary comorbidity (eg, chronic thromboembolic pulmonary hypertension, COPD > Global Initiative for Chronic Obstructive Lung Disease stage II, heart failure New York Heart Association Classification > 2, interstitial lung disease), COVID-19-associated PE, presence of comorbidities requiring intensive treatment that would interfere with the study (eg, planned surgery, malignancy requiring intense anticancer treatment), or incapability to follow study procedures or contraindications to CPET.

Patients included in the study underwent CPET within 2 to 4 weeks after index PE. All CPET was performed according to a prespecified cycle ergometer protocol including the following phases: resting, unloaded, testing, and recovery phase.^19^ Before starting the cycle ergometer protocol, spirometry was performed to calculate subsequent maximal voluntary ventilation (e-Table 1). During the testing phase, incremental exercise was performed with a ramp or minute-by-minute protocol. Exercise was continued until the point of subjective exhaustion was reached or 1 of the safety stopping criteria was met (e-Table 2). At rest and peak, capillary blood samples were obtained by finger puncture. Study procedures performed after CPET were outside of the scope of this subanalysis. The study was approved by the medical ethics review committee Medical Ethics Committee Leiden The Hague Delft (P21.103), and written informed consent was obtained from all patients before enrollment.

### Safety Analysis

To evaluate the safety of CPET shortly after acute PE, we collected data on adverse events, encompassing undesirable events occurring during or after CPET related to the index PE event or to the performance of the CPET and not caused by a preexisting, non-PE-related condition. The classification of each event as being related to CPET or the index PE event or not was determined by consensus between 2 investigators.

### CPET Analysis and Definitions

Anaerobic threshold (AT) was determined using the V-slope method.^20^ Participating sites reported outcomes of the following variables at rest, AT, and peak exercise: load, oxygen uptake ($\dot{V}o\_(2)\;$), CO_2_ output ($\dot{V}{\text{co}}_{2}$), minute ventilation (${\dot{V}}_{E}$), expiratory tidal volume, breathing frequency, heart rate (HR), oxygen pulse ($\dot{V}o_{2}$/HR), expiratory CO_2_ pressure, expiratory end-tidal CO_2_ pressure, ventilatory equivalent for CO_2_ (${\dot{V}}_{E}$/$\dot{V}{\text{co}}_{2}$), and transcutaneous oxygen saturation (Spo_2_). ECGs and BP levels were checked for abnormalities. From the capillary blood sample CO_2_ tension, lactate levels and dead space ventilation (V_D_/V_T_) were determined. Predicted values were used according to the Study of Health in Pomerania (SHIP) (e-Table 2).^21^

Maximal exercise effort was achieved at the point when the patient discontinued exercise when ≥ 1 of the following was present at peak exercise: (1) a plateau in $\dot{V}o_{2}$ (defined by an increase in $\dot{V}o_{2}$ < 2.0 mL/min/kg despite an increase in work rate by 5%-10%^19^), (2) HR > 100% predicted or HR reserve < 15 beats/min, (3) $\dot{V}e$ ≥ 85% maximal voluntary ventilation, (4) respiratory exchange ratio (VCO_2_/VO_2_) > 1.05, (5) blood lactate ≥ 8 mmol/L, and/or (6) Borg score ≥ 17 was achieved indicating severe leg discomfort or dyspnea.^19^

The following abnormal findings, defined by consensus criteria, were evaluated: peak oxygen consumption (Vo_2_peak) < 80% predicted, Δ $\dot{V}o_{2}$/Δ load ≤ 8.4 mL/min/W, ${\dot{V}}_{E}$/$\dot{V}{\text{co}}_{2}$ at AT ≥ 34, peak partial pressure of CO_2_ in the end-tidal gas > 0.3 kPa, V_D_/V_T_ > 30%, peak HR < 85% predicted, a peak oxygen pulse < 80% of the predicted value, $\dot{V}o_{2}$ at AT < 40% predicted at peak, peak Spo_2_ < 90% or > 5% drop during exercise, peak breathing frequency ≥ 60, and breathing reserve < 15%.^19^

Second, we focused on findings that reflect pulmonary vascular obstruction during exercise: insufficient cardiocirculatory reserve and alveolar perfusion defects.^22^ Stroke volume (SV) augmentation is represented by oxygen pulse and the difference between arterial and mixed venous blood oxygen content (C_a-v_O_2_):

$$\text{SV}=\text{oxygen}\;\text{pulse}/C_{a-}{\text{vO}}_{2}$$

Presuming a relatively consistent increase between rest and AT in C_a-v_O_2_ , SV augmentation is reflected by the relative increase in oxygen pulse.^23^ An oxygen pulse_AT_/oxygen pulse_rest_ < 2.6 has a 92.6% sensitivity and 66.7% specificity for stroke volume at anearobic threshold (SV_AT__)_/ stroke volume at rest (SV_rest__)_, which was 74% and 100%, respectively, for < 2.2.^24^ Thus, insufficient cardiocirculatory reserve is represented by a peak oxygen pulse < 80% and an oxygen pulse_AT_/oxygen pulse_rest_ < 2.2 and between 2.2 and 2.6. Alveolar perfusion defects are quantified by increased V_D_/V_T_ that led to ventilatory inefficiency, represented noninvasively as high ${\dot{V}}_{E}$/$\dot{V}{\text{co}}_{2}$ ratio. However, because $\dot{V}e$/$\dot{V}{\text{co}}_{2}$ reflects both Paco_2_ and V_D_/V_T_:

$${\dot{V}}_{E}/\dot{V}{\text{co}}_{2}=\text{constant}/{\text{Pa}\text{co}}_{2}\times \left(1\;-V_{D}/V_{T}\right)\text{,}$$

it can be affected by even normal ranges of Paco_2_. Specifically, ${\dot{V}}_{E}$/$\dot{V}{\text{co}}_{2}$ ≥ 34 is 78% sensitive and 80% specific for detecting V_D_/V_T_ above the lower limit of normal (0.3).^25^^,^^26^ For this reason, both ${\dot{V}}_{E}$/$\dot{V}{\text{co}}_{2}$ at AT ≥ 34 and V_D_/V_T_ > 30% were considered to represent ventilatory inefficiency.

With this, we divided patients into the following 2 groups: (1) patients with presence of any of the following: a peak oxygen pulse < 80% predicted, oxygen pulse_AT_/oxygen pulse_rest_ < 2.6, ${\dot{V}}_{E}$/$\dot{V}{\text{co}}_{2}$ at AT ≥ 34, or peak V_D_/V_T_ > 30; and (2) individuals with presence of all of the following: $\dot{V}o_{2}$ ≥ 80% predicted, peak oxygen pulse ≥ 80% predicted, oxygen pulse_AT_/oxygen pulse_rest_ ≥ 2.6, ${\dot{V}}_{E}$/$\dot{V}{\text{co}}_{2}$ at AT < 34, and peak V_D_/V_T_ ≤ 30%.

Because oxygen pulse_AT_/oxygen pulse_rest_ alone might be more specific for small stroke volume augmentation and V_D_/V_T_ alone may be more specific for ventilatory inefficiency, we performed a second analysis where group 1 was defined as with presence of oxygen pulse_AT_/oxygen pulse_rest_ < 2.6 or peak V_D_/V_T_ > 30. Group 2 was defined as presence of all of the following: (1) $\dot{V}o_{2}$ ≥ 80% predicted, (2) oxygen pulse_AT_/oxygen pulse_rest_ ≥ 2.6, and (3) peak V_D_/V_T_ ≤ 30%.

To correlate CPET outcomes with other markers of PE severity, the following markers of baseline PE severity were investigated: central, vs lobar, segmental or (sub)segmental PE; presence vs absence of RV pressure overload; and intermediate-high-risk vs low-risk PE. We defined RV pressure overload at index PE as a RV/left ventricular (LV) ratio on CTPA of ≥ 1 because echocardiography was not performed in most cases; however, if echocardiography was performed, signs of RV dysfunction on echocardiography were also included (Table 1). PE risk was classified as low, intermediate-low, or intermediate-high according to the 2019 European Society of Cardiology guideline.^2^ We correlated these subgroups to the following CPET outcomes: (1) peakVo_2_ < 80% predicted, (2) ${\dot{V}}_{E}$/$\dot{V}{\text{co}}_{2}$ at AT ≥ 34, (3) V_D_/V_T_ > 30% at peak exercise, (4) peak oxygen pulse < 80% predicted, and (5) oxygen pulse_AT_/oxygen pulse_rest_ < 2.6.

**Table 1:** Baseline Characteristics of All Included Patients (N = 100)

Characteristic	Overall
Age, y	57.8 [12.5]
Male sex	48 (48.0)
BMI, kg/m^2^	29.2 [5.9]
Unprovoked or provoked PE	
Unprovoked	68 (70.1)
Provoked by a transient risk factor	22 (22.7)
Provoked by a permanent risk factor	7 (7.2)
Comorbidities	
Previous VTE	32 (32.7)
COPD (GOLD I)	2 (2.0)
Heart failure	1 (1.0)
Hypertension	12 (12.2)
Stroke	3 (3.1)
Diabetes mellitus	5 (5.1)
Active malignancy	2 (2.0)
Anticoagulant treatment	
Direct oral anticoagulant	93 (95.9)
Low molecular weight heparin	1 (1.0)
Vitamin K antagonist	3 (3.1)
Most proximal location of PE	
Central	35 (36.5)
Lobar	12 (12.5)
Segmental	37 (38.5)
Subsegmental	12 (12.5)
RV pressure overload^a^	33 (33.7)
Hospital admission at initial presentation	61 (62.2)
sPESI	
0	77 (81.1)
≥ 1	18 (18.9)
ESC classification	
Intermediate-high risk	5 (5.3)
Intermediate-low risk	26 (27.4)
Intermediate not further classified	22 (23.2)
Low risk	42 (44.2)
MRC after 1-2 wk	
2	56 (56.0)
3	36 (36.0)
4	5 (5.0)
5	3 (3.0)
PVFS after 1-2 wk	
2	55 (55.0)
3	42 (42.0)
4	3 (3.0)

Data are presented as No. (%) or mean [SD]. Percentages are over nonmissing data. ESC = European Society of Cardiology; GOLD = Global Initiative for Chronic Obstructive Lung Disease; LV = left ventricular; MRC = Medical Research Council dyspnea scale; PE = pulmonary embolism; PVFS = Post-Venous Thromboembolism Functional Status Scale; RV = right ventricular; sPESI = simplified Pulmonary Embolism Severity Index.

a RV pressure overload at index PE was defined as a RV/LV ratio on CT pulmonary angiography of ≥ 1 because echocardiography was not performed in most cases; however, if echocardiography was performed, any of the following findings were also classified as having RV pressure overload: (1) RV/LV end-diastolic diameter ratio ≥ 0.9 (apical or subcostal 4-chamber view), (2) RV end-diastolic diameter > 30 mm (parasternal long-axis or short-axis view), (3) RV free wall hypokinesis (any view), (4) tricuspid regurgitation velocity > 2.8 m/s (apical or subcostal 4-chamber view, or parasternal short-axis view), or (5) inferior vena cava diameter > 21 mm with decreased inspiratory collapse (< 50% with a sniff or < 20% with quiet inspiration).

### Statistical Analysis

Categorical variables are presented as frequency with percentages, and variable data are presented as median with interquartile range.

We calculated ORs for markers of PE severity on odds of having (1) Vo_2_peak < 80% predicted, (2) ${\dot{V}}_{E}$/$\dot{V}{\text{co}}_{2}$ at AT ≥ 34, (3) V_D_/V_T_ > 30% at peak exercise, (4) peak oxygen pulse < 80% predicted, or (5) oxygen pulse_AT_/oxygen pulse_rest_ < 2.6. To visualize overlap of markers associated with pulmonary vascular disease, we plotted these in a Venn diagram. All analyses were performed using R version 4.3.1 (R Foundation for Statistical Computing).

## Results

### Patient Population

We included the first 100 patients with acute PE enrolled in the PE@HOME study who underwent CPET 2 to 4 weeks after diagnosis (e-Fig 1). Mean age was 58 years, and 48% were male. Of all patients, 12% had hypertension, 33% had previous VTE, 2% had COPD (Global Initiative for Chronic Obstructive Lung Disease stage I or II), and 2% had active malignancy (Table 1). All patients had a hemodynamically stable acute PE at presentation. The index PE presented in most patients as unprovoked (70%). Approximately one-half of the patients had PE in a segmental or subsegmental location (38.5% and 12.5%, respectively). Most patients had no signs of RV pressure overload (66%).

### Cardiopulmonary Exercise Testing

CPET was performed after a median of 20 days (interquartile range, 20-27). Six patients had CPET > 28 days post-PE due to logistical issues. In 22 of 100 patients, capillary blood gas analysis could not be performed. Exercise was continued to exhaustion (ie, the patient requested to stop exercise because of the subjective opinion they could not perform any more exercise) in 98 patients without incident. In 2 patients, CPET was stopped because of systolic BP > 250 mm Hg before subjective exhaustion; hypertension was preexisting and deemed to be non-PE related. In another patient, CPET was continued until subjective exhaustion was achieved, but retrospective evaluation of the ECG showed slight ST elevations in V_4_ to V_6_ during exercise without presence of chest pain; subsequent echocardiography showed no signs of PH and a nondilated right ventricle. Repeat CPET showed no ECG abnormalities, therefore not representing an adverse event.

Variable and parameter CPET data are depicted in Table 2 and e-Table 3. No patients had a submaximal test (because in the 3 patients in whom exercise was stopped before subjective exhaustion was reached, postexercise analysis showed that the criteria for performing maximal exercise effort was achieved). Generally, peak aerobic exercise capacity was preserved. Indeed, 77% of the patients had a Vo_2_peak ≥ 80% predicted.

**Table 2:** Cardiopulmonary Exercise Testing Data 2 to 4 wk After Pulmonary Embolism

Variables	Median (IQR)	Abnormal if	Frequency of Abnormality (%)
Metabolic			
Peak load, W	140 (104-182)		
Vo_2_peak, mL/min	1,787 (1,463-2,173)	Vo_2_peak < 80% predicted	23 (23)
Δ $\dot{V}o_{2}$/Δ load, mL/min/W	10 (9.4-11)	Δ $\dot{V}o_{2}$/Δ load ≤ 8.4 mL/min/W	10 (10)
Peak RER	1.12 (1.05-1.18)		
Borg score for dyspnea at peak	16 (13-17)	Borg score for dyspnea at peak ≥ 17	34 (35)
Borg score for exhaustion at peak	15 (13-17)	Borg score for exhaustion at peak ≥ 17	40 (40)
Cardiopulmonary			
Ventilatory inefficiency			
${\dot{V}}_{E}$/$\dot{V}{\text{co}}_{2}$ at AT	31.1 (28.4-34.3)	${\dot{V}}_{E}$/$\dot{V}{\text{co}}_{2}$ at AT ≥ 34	28 (28)
		${\dot{V}}_{E}$/$\dot{V}{\text{co}}_{2}$ at AT above the age and sex specific cutoff^21^	48 (48)
Peak P(c-ET)CO_2_	0.4 (0.15-0.713)	P(c-ET)CO_2_ at maximum > 0.3 kPa	47 (56)
Peak V_D_/V_T_, %	27 (20.8-32.2)	Peak V_D_/V_T_ > 30%	27 (27)
Cardiocirculatory			
Peak HR, beats/min	146 (136-164)	Peak HR < 85% predicted	15 (15)
Peak oxygen pulse ($\dot{V}o_{2}$/HR), mL/beat	11.8 (10.1-14)	Peak oxygen pulse < 80% predicted	27 (35)
		Oxygen pulse_AT_/oxygen pulse_rest_ < 2.2	46 (46)
		Oxygen pulse_AT_/ oxygen pulse_rest_ ≥ 2.2 and ≤ 2.6	27 (27)
$\dot{V}o_{2}$ at AT, mL/min	1050 (856-1,281)	$\dot{V}o_{2}$ at AT < 40% predicted at peak	10 (10)
Ventilatory			
Peak ${\dot{V}}_{E}$, L/min	73.4 (61.1-90)		
Peak oxygen saturation, %	96 (94-98)	Peak Spo_2_ < 90% or > 5% drop during exercise	4 (4)
Peak breathing frequency, breaths/min	37.2 (32.3-42.2)	Peak breathing frequency ≥ 60 breaths/min	1 (1)
Peak breathing reserve, (MVV − ${\dot{V}}_{E}$)/MVV), %	36.3 (27.6-43.1)	BR < 15%	7 (7)

Variable data are reported as median (IQR), and parameter data are reported as a frequency (%). AT = anaerobic threshold; BR = breathing rate; HR = heart rate; IQR = interquartile range; MVV = maximal voluntary ventilation; p(c-ET)CO_2_ = partial pressure of CO_2_ in the end-tidal gas; RER= respiratory exchange ratio; Spo_2_ = transcutaneous oxygen saturation; V_D_/V_T_ = dead space to tidal volume ratio; ${\dot{V}}_{E}$/$\dot{V}{\text{co}}_{2}$ = ventilatory equivalent for CO_2_; $\dot{V}o_{2}$ = oxygen uptake; Vo_2_peak = peak oxygen consumption.

Twenty-eight patients had ${\dot{V}}_{E}$/$\dot{V}{\text{co}}_{2}$ at AT ≥ 34 (28%), and 27 had peak V_D_/V_T_ > 30% (35%). Twenty-seven patients had peak oxygen pulse < 80% predicted (27%), 46 had an oxygen pulse_AT_/oxygen pulse_rest_ < 2.2 (46%), and 27 had an oxygen pulse_AT_/oxygen pulse_rest_ ≥ 2.2 and < 2.6 (27%).

Forty-seven patients had peak partial pressure of carbon dioxide in the end-tidal gas > 0.3 kPa (47%). Fifteen patients had a peak HR < 85% predicted (15%), and 10 patients had a $\dot{V}o_{2}$ at AT < 40% predicted (10%). Only 4 patients had a peak Spo_2_ < 90% or > 5% drop during exercise (4%), 1 patient had a peak breathing frequency ≥ 60 (1%), and 7 patients had a breathing reserve < 15% (7%).

**Figure 1:**
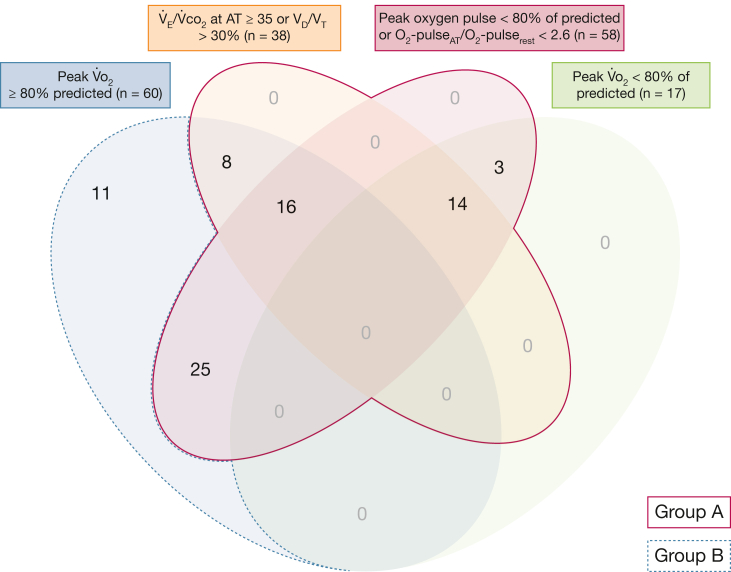
Overview overlap patterns of limitation. The Venn diagram presents the absolute number per category. Patients in the blue circle had a Vo_2_peak ≥ 80% predicted. Patients in yellow had a ${\dot{V}}_{E}$/$\dot{V}{\text{co}}_{2}$ ≥ 34 at AT or a peak V_D_/V_T_ > 30%. Patients in red had a peak oxygen pulse < 80% predicted or an oxygen pulse_AT_/oxygen pulse_rest_ < 2.6. Patients in green had a Vo_2_peak < 80% predicted. Patients outside of these circles do not have these characteristics. Overlap of circles means multiple characteristics are present. AT = anaerobic threshold; V_D_/V_T_ = dead space to tidal volume ratio; ${\dot{V}}_{E}$/$\dot{V}{\text{co}}_{2}$ = ventilatory equivalent for CO_2_; Vo_2_peak = peak oxygen consumption.

Figure 1 presents the overlap of variables sensitive for pulmonary vascular disease. Of the 77 patients included in this subanalysis, 66 were classified as group 1 (86%) and 11 were classified as group 2 (14%). Interestingly, there were no patients with a $\dot{V}o_{2}$ < 80% predicted that had no abnormalities in markers sensitive for pulmonary vascular disease (ie, peak oxygen pulse < 80% predicted, oxygen pulse_AT_/oxygen pulse_rest_ < 2.6, ${\dot{V}}_{E}$/$\dot{V}{\text{co}}_{2}$ at AT ≥ 34 or peak V_D_/V_T_ > 30). e-Table 6 shows that in group 2, no patients had a drop in Spo_2_, and 4 had partial pressure of CO_2_ in the end-tidal gas at maximum > 0.3 kPa of which 2 patients were < 0.66 kPa (5 mm Hg is also a frequently used cutoff).

For the more specific analysis only including Vo_2_peak < 80% predicted, oxygen pulse_AT_/oxygen pulse_rest_ < 2.6, and a peak V_D_/V_T_ > 30%, similar numbers were seen: group 1 included 65 patients (84%) and group 2 included 11 patients (14%). In 1 patient there was presence of a Vo_2_peak < 80% predicted but no signs of an oxygen pulse_AT_/oxygen pulse_rest_ < 2.6 or peak V_D_/V_T_ > 30%.

### Markers of PE Severity

Table 3 presents the association between markers of PE severity and CPET outcomes, e-Table 4 presents the parameter CPET data per subgroup of all variables. Presence of RV pressure overload was not associated with increased incidence of abnormal CPET outcomes. Central PE was associated with an increased incidence of a Vo_2_peak < 80% predicted and a peak oxygen pulse < 80% predicted compared with a lobar or (sub)segmental PE (OR, 3.4; 95% CI, 1.1-10 and OR, 3.4; 95% CI, 1.2-9.7, respectively).

**Table 3:** OR of Markers of Pulmonary Embolism Severity

	Vo_2_peak < 80% Predicted		Peak Oxygen Pulse < 80% Predicted		Oxygen Pulse_AT_/Oxygen Pulse_rest_ < 2.6		${\dot{V}}_{E}$/$\dot{V}{\text{co}}_{2}$ at AT ≥ 34		Peak V_D_/V_T_ > 30%	
	No. (%)	OR (95% CI)	No. (%)	OR (95% CI)	No. (%)	OR (95% CI)	No. (%)	OR (95% CI)	No. (%)	OR (95% CI)
RV pressure overload (n = 33)	9 (27)	1.4 (0.45-4)	10 (30)	1.2 (0.43-3.4)	28 (85)	2.7 (0.86-10)	11 (33)	1.5 (0.53-4.1)	7 (21)	0.79 (0.23-2.5)
No RV pressure overload (n = 65)^a^	14 (22)		17 (26)		43 (66)		16 (25)		19 (29)	
Central (n=35)	13 (37)	3.4 (1.1-10)	15 (43)	3.4 (1.2-9.7)	28 (80)	1.8 (0.63-5.9)	10 (29)	1.1 (0.38-3)	10 (29)	1.1 (0.38-3.4)
Lobar-(sub)segmental (n = 61)^a^	9 (15)		11 (18)		41 (67)		16 (26)		15 (25)	
Intermediate-high (n = 5)	3 (60)	10 (0.94-150)	4 (80)	14 (1.17-740)	5 (100)	4.6 (1.5-15)	4 (80)	18 (1.5-980)	0 (0)	0 (0-3.7)
Low risk (n = 42)^a^	5 (12)		9 (21)		24 (57)		7 (17)		7 (21)	

AT = anaerobic threshold; RV = right ventricular; V_D_/V_T_ = dead space to tidal volume ratio; ${\dot{V}}_{E}$/$\dot{V}{\text{co}}_{2}$ = ventilatory equivalent for CO_2_; Vo_2_peak = peak oxygen consumption.

a Reference subgroup.

Patients with an intermediate- high risk PE had an increased incidence of a Vo_2_peak < 80% predicted, peak oxygen pulse < 80%, oxygen pulse_AT_/oxygen pulse_rest_ < 2.6, and ${\dot{V}}_{E}$/$\dot{V}{\text{co}}_{2}$ at AT ≥ 34, (OR, 10; 95% CI, 0.94-150; OR, 14; 95% CI, 1.2-740; OR, 4.6; 95% CI, 1.5-15; OR, 18; 95% CI, 1.5-980, respectively) compared with patients with low-risk PE.

## Discussion

Our results indicate that strenuous exercise is safe and well tolerated as soon as 2 to 4 weeks after acute PE not associated with hemodynamic shock. For the interpretation of these findings, we applied the following definitions: a reduced exercise capacity was defined as $\dot{V}o_{2}$ < 80% predicted, ventilatory inefficiency was defined as ${\dot{V}}_{E}$/$\dot{V}{\text{co}}_{2}$ at AT ≥ 34 or peak V_D_/V_T_ > 30%, and insufficient cardiocirculatory reserve was defined as a peak oxygen pulse < 80% predicted and oxygen pulse_AT_/oxygen pulse_rest_ < 2.6. Among patients with no signs of a reduced exercise capacity, no signs of ventilatory inefficiency, and no signs of insufficient cardiocirculatory reserve, we concluded that they had an adequate cardiopulmonary response during exercise (patients in group 2). Using these definitions, despite reporting dyspnea, 77% of patients had a normal exercise capacity (ie, normal Vo_2_peak) and 14% of patients had no signs for a reduced exercise capacity ($\dot{V}o_{2}$ < 80% predicted), no signs of ventilatory inefficiency (${\dot{V}}_{E}$/$\dot{V}{\text{co}}_{2}$ at AT ≥ 34 and peak V_D_/V_T_ > 30%), and no signs of insufficient cardiocirculatory reserve (peak oxygen pulse < 80% predicted and oxygen pulse_AT_/oxygen pulse_rest_ < 2.6). The presentation of acute PE was only partially predictive of post-PE cardiopulmonary function during CPET. Patients diagnosed with anatomically central PE had an increased incidence of Vo_2_peak < 80% predicted, peak oxygen pulse < 80% predicted, and oxygen pulse_AT_/oxygen pulse_rest_ < 2.6. Those with intermediate-high risk PE appeared to have a higher risk of peak oxygen pulse < 80% predicted, oxygen pulse_AT_/oxygen pulse_rest_ < 2.6, or ${\dot{V}}_{E}$/$\dot{V}{\text{co}}_{2}$ at AT ≥ 34.

Up to one-half of patients with acute PE experience persistent symptoms and limitations in daily life despite anticoagulation, which is one aspect of post-PE syndrome.^5^, ^6^, ^7^, ^8^, ^9^, ^10^ One potential cause is post-PE functional impairment where fear of complications combined with cautious medical advice for resuming exercise results in inactivity and deconditioning.^11^^,^^27^, ^28^, ^29^ Early exercise training has been suggested as a method to reduce inactivity and prevent deconditioning, potentially mitigating post-PE syndrome.

Our results demonstrated that performing exercise in selected patients with acute PE 2 to 4 weeks after diagnosis was safe, with no PE-related adverse events. Because CPET involves higher intensity than typical exercise training, our study suggests the potential safety of early initiation of exercise training and aligns with previous studies reporting no adverse events during such programs for patients with PE.^11^^,^^16^^,^^30^^,^^31^ In contrast, an excessive caution regarding exercise resumption may contribute to inactivity and deconditioning. However, our study focused on a selected group of hemodynamically stable patients, and the safety of exercise at home and in patients with severe acute PE remains to be established.

Despite all patients still experiencing dyspnea and functional limitations 1 to 2 weeks after diagnosis, 1 of 7 patients displayed no signs of reduced exercise capacity, inefficient ventilation, or insufficient cardiocirculatory reserve of which we conclude had an adequate cardiopulmonary response during exercise. However, there may be discussion on the definition of adequate cardiopulmonary response during exercise as decreasing Spo_2_ or abnormal partial pressure of CO_2_ in the end-tidal gas at maximum are not included. Nonetheless, if these parameters would be included, similar results would be found because 1 of 9 patients would have an adequate cardiopulmonary response during exercise. Going into depth, out of the 66 patients with signs of ventilatory inefficiency or signs of insufficient cardiocirculatory reserve, 49 were still able to achieve a normal Vo_2_peak (74%). This important finding has 2 implications: (1) Vo_2_peak alone is insensitive for ruling out cardiopulmonary limitations (eg, exercise-related ventilatory inefficiency, poor cardiac reserve after PE) and (2) similar to other pulmonary diseases, patients after PE may safely exercise of the type experienced during CPET despite the presence of demonstrable physiological defects associated with dyspnea because maximum exercise was achieved without the occurrence of undesired events; however, long-term outcomes remain to be investigated.

Previous studies have reported that 47% to 55% of patients exhibit abnormal exercise capacity and 50% display adequate cardiopulmonary reserve 3 to 12 months after PE.^5^^,^^15^ Moreover, when only looking at patients with post-PE impairment, approximately 20% exhibit adequate cardiopulmonary response during follow-up.^15^ When looking at persistent symptoms and persistent vascular obstruction on imaging, the same pattern is seen: there is an association between persistent symptoms after PE and persistent vascular obstruction, but a large proportion of patients with persistent symptoms do not have persistent vascular obstruction.^32^ Therefore, these results highlight that not all post-PE dyspnea and/or functional limitation can be explained by solely inadequate cardiopulmonary response and/or abnormal exercise capacity. In these patients, the cause of their post-PE symptoms remains unclear. The sensation of dyspnea in patients after PE may be related to a neuromechanical dissociation and influenced by persistent clots and ventilation perfusion mismatch.^33^ Consequently, a given respiratory work load can result in a different perception of dyspnea in various individuals.^34^ Psychological factors, notably anxiety, may be involved in the sensation of dyspnea. To our knowledge, clear results from physiological studies explaining the cause of dyspnea in patients after PE are lacking and beyond the scope of this study.

On the other hand, in 86% of patients with dyspnea and functional limitations, we did observe an abnormal cardiopulmonary limitation when performing exercise, and during acute PE follow-up similar numbers are reported.^5^^,^^15^ Patients with a central PE or intermediate-high risk PE are at increased risk for such abnormalities. The association of intermediate-high risk with abnormal exercise capacity was nonsignificant, likely due to statistical power limitations (because the relationship between intermediate risk and abnormal exercise capacity was significant) (e-Table 5).

The presence of RV pressure overload at presentation did not seem to correlate with abnormalities on CPET, nor did V_D_/V_T_ correlate with any of the markers of PE severity. Moreover, we expected that RV pressure overload would be within the causal pathway of central PE leading to RV pressure overload, resulting in abnormalities on CPET because central PE is associated with increased clot burden, and increased clot burden is associated with increased RV/LV ratio on CTPA.^35^ However, we observed no association between RV pressure overload and abnormalities on CPET, which could be attributed to the fact that we did not assess RV pressure overload quantitatively, but only qualitatively measured the RV/LV ratio as a surrogate marker for cardiac dysfunction.

It remains unclear whether patients with abnormal cardiovascular limitations in this cohort are the ones who will experience persistent symptoms or how exercise training programs influence post-PE syndrome. The ongoing PE@HOME trial—of which this study was a preplanned subanalysis—is currently investigating these questions.

The study’s multicenter prospective design and the novelty of performing CPET soon after PE diagnosis are key strengths. However, several limitations should be noted. First, as part of the ongoing PE@HOME trial, only a selected group of patients with acute PE was included, excluding those unable to participate in an 8-week exercise program (eg, those undergoing cancer treatment, pregnant, discharged to rehabilitation). Second, capillary blood samples, rather than arterial Paco_2_, were used to calculate V_D_/V_T_, possibly overestimating abnormalities; however, the bias (< 1 mm Hg) is unlikely to affect results.^36^ Third, despite the inclusion of a relatively large number of patients, the subgroup analyses may have been underpowered. We acknowledge potential limitations, including the lack of data on hospitalization duration, which could impact in-hospital deconditioning. However, because none of the participants were critically ill and most (94.7%) were at intermediate-low or low risk, the influence of deconditioning because of hospitalization is likely to be minimal. Also, patients were enrolled based on dyspnea and functional limitations, confirmed 1 to 2 weeks after diagnosis; however, some improvement may have occurred before CPET at 2 to 4 weeks after PE. Finally, although a standardized CPET protocol was followed, minor locoregional variations might exist.

## Interpretation

In conclusion, strenuous exercise in the first weeks after acute PE was feasible and well tolerated. The discrepancy between subjective symptoms and objective limitations suggests that post-PE symptoms are multifactorial. In this study, centrally located emboli and intermediate-high risk PE may lead to a higher risk of detecting CPET abnormalities.

## Funding/Support

This work was supported by a grant from The Dutch Thrombosis Association (project number 2021-B).

## Financial/Nonfinancial Disclosures

None declared.

## Figures and Tables

**Supplementary Material:**
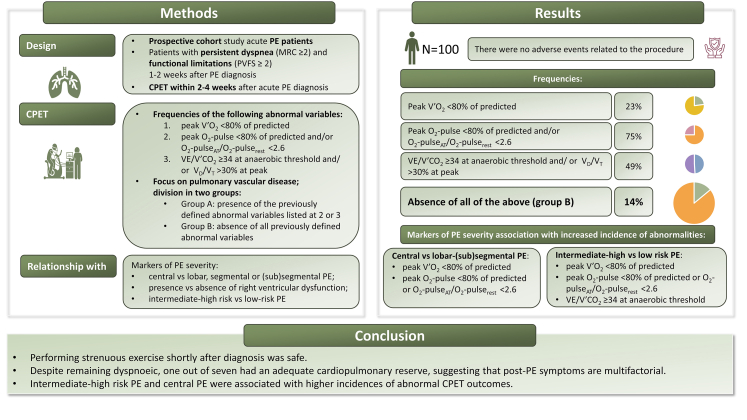


## Supplementary Data

e-Online Data
