# Supplementary material for: Cardiopulmonary Exercise Testing in People With Dyspnea With a Recent Acute Pulmonary Embolism
Source: CHEST Pulm. 2025 Mar 18;3(3):100164. doi: 10.1016/j.chpulm.2025.100164 (PMC13417568; doi:10.1016/j.chpulm.2025.100164)
Supplement: e-Online Data [file mmc1.docx]

Supplementary file to “Cardiopulmonary exercise testing in dyspnoeic persons with a recent acute pulmonary embolism”

Dieuwke Luijten^1^, Josien van Es^2,3^, Jannie J. Abbink^4^, Stefano Barco^5^, Johanna M.W. van den Berg^6^, Waleed Ghanima^7^, Menno V. Huisman^1^, Coen van Kan^2,3^, Bas Langeveld^8^, Ivo van der Lee^9^, Rosa Mali^1, 10^, Thijs E. van Mens^1^, Timothy A. Morris^11^, Maria Overbeek^12^, Mart van der Plas^12^, Martijn A. Spruit^13, 14^, Frederikus A. Klok^1^, Anton Vonk Noordegraaf^2^, and Maarten K. Ninaber^10^

^1^ Department of Medicine - Thrombosis and Hemostasis, Leiden University Medical Center, Leiden, The Netherlands

^2^ Department of Pulmonology, Amsterdam UMC, VU University Medical Centre, Amsterdam, The Netherlands

^3^ Department of Pulmonology, OLVG, Amsterdam, The Netherlands

^4^ Basalt rehabilitation Center, Leiden, The Netherlands

^5^ Department of Angiology, University Hospital of Zurich, Zurich, Switzerland

^6^ Department of Pulmonology, Haga Teaching Hospital, The Hague, The Netherlands

^7^ Departments of Oncology, Medicine and Research Ostfold Hospital Trust Kalnes, Norway

^8^ Department of Pulmonology, Deventer ziekenhuis, Deventer, The Netherlands

^9^ Department of Pulmonology, Spaarne gasthuis, Haarlem, The Netherlands

^10^ Department of Pulmonology, Leiden University Medical Center, Leiden, the Netherlands

^11^ Division of Pulmonary and Critical Care Medicine, University of California at San Diego, La Jolla, CA, USA.

^12^ Department of Pulmonology, Haaglanden Medisch Centrum, The Hague, The Netherlands

^13^ Department of Research & Development, Ciro, Horn, the Netherlands

^14^ Department of Respiratory Medicine, Maastricht University Medical Centre, NUTRIM Institute of Nutrition and Translational Research in Metabolism, Faculty of Health, Medicine and Life Sciences, Maastricht, The Netherlands.

Contents

[Table S1: Variables 3](#_Toc184290812)

[Table S2: stopping criteria CPET 3](#_Toc184290813)

[Figure S1 flow chart of study inclusions 4](#_Toc184290814)

[Table S3. Variable CPET data per subgroup 5](#_Toc184290815)

[Table S4. Parameter CPET data per subgroup 6](#_Toc184290816)

[Table S5: OR of intermediate vs low risk PE 7](#_Toc184290817)

[Table S6. Overview CPET data in group B 8](#_Toc184290818)

# Table S1: Variables

| **Variable [unit]** | **Timepoint** | **Formula** | **Abnormal if** |
| --- | --- | --- | --- |
| **Predicted variables** | | |  |
| increment wattage per minute | n.a. | predicted V’O2 at peak exercise V’O2 during unloaded phase)/100)^α^ |  |
| V’O2 [mL/min] | Peak  AT | 4307 -241 *AG -20 * AG^2^ -1281*S + 133*AG*S [34]  1841.723 - 45.943 *AG -3.6219*AG^2^ -411.439*S + 15.3597*AG*S [34] | <80% predicted |
| Load [wattage] | Peak | For males: –103.512 – 1.5576 *A + 2.2114 *H–0.1198*W [21]  For females: –80.628 –0.7698*A+1.4038*H+0.2873*W [21] |  |
| HR | Peak | For males: 202.986 – 0.8214 * A + 0.0796 *H–0.1400 *W [21]  For females: 214.524 – 0.7549 *A–0.0230 *H–0.1864 *W [21] | <80% predicted |
| V’E/V’CO2 | Rest  AT | For males: 12.136 + 0.1665 * A+ 0.0904 *H–0.0298 *W[21]  For females: 8.338 + 0.0212 * A+ 0.1380 *H–0.0086 *W[21]  For males: 25.288 + 0.1203 *A–0.0387 *H+ 0.0193 *W[21]  For females: 8 25.447 + 0.0090 *A–0.0120 *H+ 0.0346 *W [21] | ≥ 35 |
| O2-pulse | Peak | For males: 1.475 – 0.0613 *A + 0.0621 *H+ 0.0724 *W [21]  For females: –1.720 – 0.0008 *A + 0.0512 *H+ 0.0547 *W [21] | <80% predicted |
| V_D_/V_T_ [%] | Peak | For males: –0.223+0.0026*A+0.0013*H+0.0001*W [21]  For females: –0.194+0.0026*A+0.0013*H+0.0001*W [21] |  |
| **Calculated variables** | | |  |
| V_D_/V_T_ [%] | Peak | ((PcCO2 – PECO2) / PaCO2) – physiological dead space from mask and turbine | >30% |
| MVV | n.a. | FEV1 * 40 |  |

^α^ deviation from this formula based on daily activities and exercise was allowed; A age in years; AG age group (25–34=1; 35–44=2; 45–54=3; 55–64=4; ≥65=5); BMI body mass index groups (≤25 kg/m2=0; >25 kg/m2=1); S sex (males=1; females=2); H height in centimeters; W weight in kilograms;

# Table S2: stopping criteria CPET

| 1. Chest pain appropriate to ischemia. |
| --- |
| 1. Ischemic ECG changes. |
| 1. Complex ectopy (propagated ventricular extrasystoles, particularly with an R-on-T pattern and runs of 3 or more extrasystoles). |
| 1. Emergence of 2nd or 3rd degree AV block. |
| 1. Decrease in systolic blood pressure > 20 mmHg from the highest value during the test. |
| 1. Hypertension (> 250 mmHg systolic; > 120 mmHg diastolic). |
| 1. Severe desaturation: SpO2 = 80% plus symptoms or signs of severe hypoxaemia (blue lips/fingers, headache, dizziness). |
| 1. Sudden pallor. |
| 1. Loss of coordination. |
| 1. Mental confusion |
| 1. Dizziness or fainting. |
| 1. Signs of respiratory failure (hypoxaemia, hypercapnia, central cyanosis (blue lips), headache, dizziness, severe wheezing). |
| 1. Patient was unable to continue exercise at 60 rounds/minute |

# Figure S1 flow chart of study inclusions


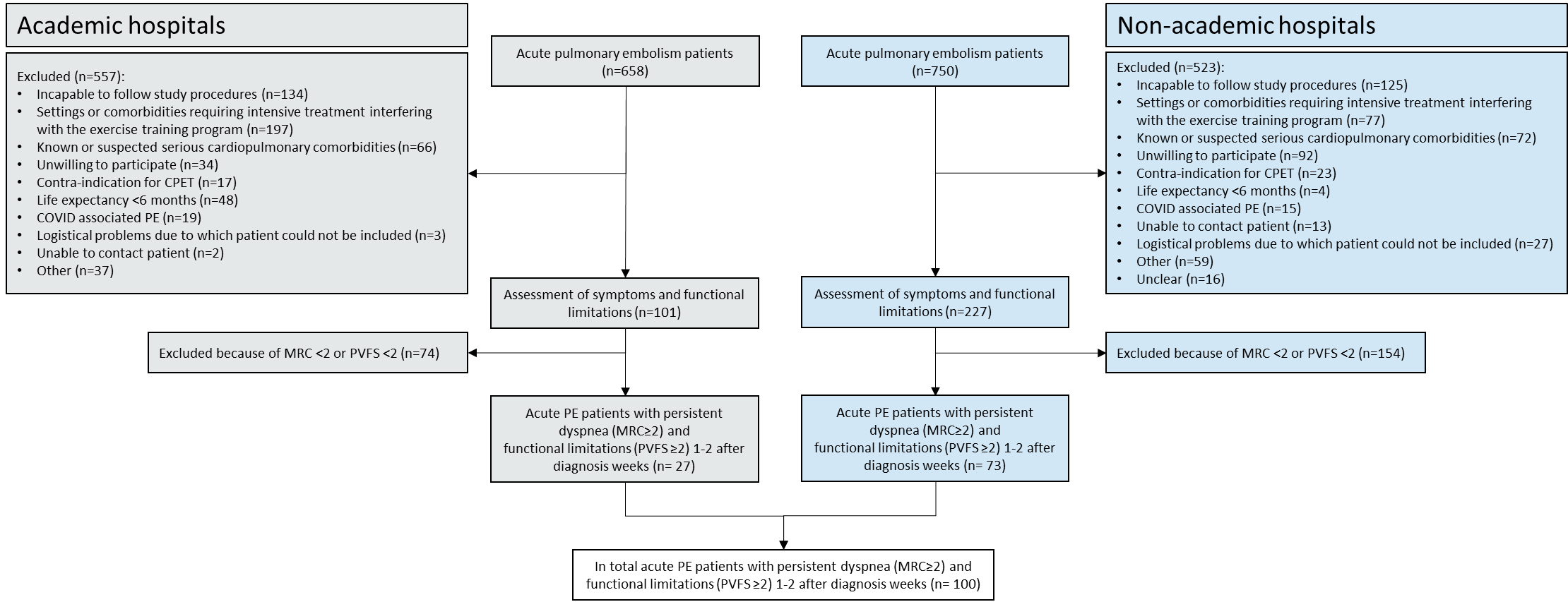


| Table S3. Variable CPET data per subgroup | | | | | | | | | | | |
| --- | --- | --- | --- | --- | --- | --- | --- | --- | --- | --- | --- |
| Median (IQR) | Overall sub-acute CPET | No RVD at baseline PE | RVD at baseline PE | Central PE | Lobar or (sub)segmental PE | low risk PE | Intermediate risk PE | Intermediate-high risk PE | Abnormal troponin | No abnormal troponin | |
| Number of patients included | 100 | 65 | 33 | 35 | 61 | 42 | 53 | 5 | 34 | 20 | |
| Metabolic |  |  |  |  |  |  |  |  |  |  | |
| Rest |  |  |  |  |  |  |  |  |  |  | |
| V’O2 [mL/min] | 362 (304-416) | 358 (280-417) | 383 (335-406) | 383 (320-415) | 355 (280-406) | 362 (286-418) | 364 (314-406) | 406 (386-462) | 367 (305-412) | 374 (274-406) | |
| RER | 0.83 (0.79-0.89) | 0.83 (0.79-0.9) | 0.83 (0.8-0.86) | 0.83 (0.79-0.86) | 0.83 (0.8-0.9) | 0.855 (0.8-0.9) | 0.81 (0.79-0.86) | 0.8 (0.8-0.82) | 0.815 (0.792-0.858) | 0.835 (0.788-0.892) | |
| AT |  |  |  |  |  |  |  |  |  |  | |
| V’O2 [mL/min] | 1050 (856-1281) | 1045 (820-1344) | 1110 (901-1207) | 1019 (878-1218) | 1080 (827-1344) | 1172 (855-1641) | 991 (863-1200) | 892 (866-900) | 932 (864-1174) | 991 (800-1255) | |
| RER | 0.86 (0.79-0.91) | 0.86 (0.788-0.915) | 0.86 (0.8-0.89) | 0.86 (0.8-0.905) | 0.855 (0.788-0.91) | 0.87 (0.79-0.91) | 0.85 (0.79-0.9) | 0.88 (0.87-0.89) | 0.865 (0.8-0.908) | 0.86 (0.775-0.89) | |
| Load [watt] | 55 (40-86) | 60 (45-97) | 54 (40-73) | 52 (40-72) | 62 (40-97) | 73 (48-107) | 50 (39-73) | 40 (30-40) | 47 (39-68) | 48 (42-71) | |
| Peak |  |  |  |  |  |  |  |  |  |  | |
| V’O2 [mL/min] | 1787 (1463-2173) | 1795 (1465-2178) | 1710 (1462-2147) | 1764 (1410-1940) | 1830 (1491-2253) | 1887 (1613-2251) | 1616 (1320-2022) | 1407 (1279-1463) | 1563 (1289-1922) | 1686 (1410-2173) |  |
| RER | 1.12 (1.05-1.18) | 1.12 (1.05-1.18) | 1.13 (1.05-1.17) | 1.13 (1.02-1.16) | 1.12 (1.06-1.18) | 1.13 (1.08-1.19) | 1.12 (1.04-1.17) | 1.14 (1.12-1.15) | 1.12 (1.04-1.17) | 1.12 (1.05-1.2) | |
| Load [watt] | 140 (104-182) | 142 (113-180) | 132 (98-180) | 121 (102-157) | 150 (110-200) | 153 (120-195) | 120 (92-172) | 90 (80-92) | 119 (90-162) | 120 (100-169) | |
| Cardiovascular/ gas exchange |  |  |  |  |  |  |  |  |  |  | |
| Rest |  |  |  |  |  |  |  |  |  |  | |
| HR [beats/min] | 80 (70-95) | 81 (71-95) | 77 (69-87) | 80 (71-95) | 79 (69-92) | 80 (68-92) | 80 (71-96) | 81 (77-96) | 78 (71-94) | 82 (72-95) | |
| V’O2 /HR (O2 pulse) [mL/beat] | 4.6 (3.5-5.43) | 4.5 (3.4-5.4) | 4.8 (4-5.5) | 4.8 (3.8-5.5) | 4.5 (3.4-5.3) | 4.8 (3.42-5.5) | 4.5 (3.6-5.3) | 4.8 (4.2-5.4) | 4.55 (3.8-5.4) | 4.7 (3.85-5.2) | |
| Vd/Vt | 26.4 (21-33) | 26.4 (21-32.5) | 25.9 (16.8-33) | 24.5 (14.5-31.5) | 26 (21.6-33) | 25.7 (21-30.6) | 27 (19.5-33.5) | 26 (19.8-30.8) | 28 (13-33) | 25.5 (18.8-32.8) | |
| P(ET-E)CO2 | 1.1 (0.9-1.25) | 1.1 (0.9-1.3) | 1.07 (0.89-1.18) | 1.05 (0.8-1.2) | 1.12 (1-1.27) | 1.1 (0.9-1.31) | 1.09 (0.933-1.2) | 1.13 (1.12-1.25) | 1.1 (0.931-1.23) | 1.08 (0.9-1.21) | |
| P(c-ET)CO2 | 0.83 (0.5-1.17) | 0.815 (0.507-1.09) | 0.897 (0.5-1.2) | 0.8 (0.41-1.2) | 0.825 (0.502-1.15) | 0.825 (0.548-0.962) | 0.848 (0.44-1.26) | 0.09 (-0.47-0.467) | 0.79 (0.29-1.2) | 0.93 (0.8-1.01) | |
| AT |  |  |  |  |  |  |  |  |  |  | |
| HR [beats/min] | 105 (96-117) | 108 (98-119) | 103 (94-113) | 103 (94-114) | 110 (97-124) | 110 (98-117) | 104 (97-123) | 104 (97-122) | 102 (96-117) | 104 (98-121) | |
| V’O2 /HR (O2 pulse) [mL/beat] | 9.9 (8.3-12) | 9.75 (7.8-12) | 10.3 (8.7-12.3) | 9.8 (8.7-11.4) | 10.2 (7.68-12.4) | 10.8 (8.7-13) | 9.3 (8.2-11.3) | 8.7 (7.7-9.2) | 9.55 (8.25-11.3) | 9.6 (8-11.2) | |
| V’E/V’CO2 | 31.1 (28.4-34.3) | 30.9 (27.2-44) | 31.5 (29.3-35.1) | 31.1 (29.6-35) | 30.4 (27-34.1) | 29.4 (26.9-32.7) | 32.2 (29.4-36.8) | 36.9 (36.8-37) | 32.4 (29.2-46.4) | 31.3 (27-33.5) | |
| Peak |  |  |  |  |  |  |  |  |  |  | |
| HR [beats/min] | 146 (136-164) | 147 (136-171) | 145 (132-153) | 145 (126-154) | 147 (137-169) | 148 (137-169) | 145 (130-157) | 147 (120-152) | 142 (127-152) | 146 (138-160) | |
| V’O2 /HR (O2 pulse) [mL/beat] | 11.8 (10.1-14) | 11.6 (10.2-13.9) | 11.9 (10-14.3) | 11.8 (9.95-13) | 11.8 (10.2-14.8) | 12.4 (10.6-15) | 11.6 (9.7-13.3) | 9.8 (9.5-10) | 11.6 (9.72-13.3) | 11.2 (9.32-13) | |
| Vd/Vt [%] | 27 (20.8-32.2) | 27 (20-33.9) | 27 (21-31) | 26.5 (20.8-31) | 27 (18-32.8) | 26 (20-33) | 27 (20.5-32) | 22 (18.5-25.5) | 28 (20.5-32) | 26 (14.2-28.8) | |
| P(ET-E)CO2 | 1.1 (0.97-1.2) | 1.1 (1.01-1.3) | 1.07 (0.931-1.2) | 1.04 (0.936-1.11) | 1.16 (1-1.28) | 1.1 (1.01-1.2) | 1.08 (0.934-1.2) | 0.94 (0.83-0.96) | 1.08 (0.931-1.23) | 1.07 (1.02-1.13) | |
| P(c-ET)CO2 | 0.4 (0.15-0.713) | 0.4 (0.15-0.92) | 0.32 (0.172-0.62) | 0.4 (0.2-0.64) | 0.3 (0.134-0.89) | 0.36 (0.147-0.915) | 0.4 (0.142-0.725) | 0.25 (0.232-0.343) | 0.4 (0.217-0.685) | 0.33 (0.15-0.83) | |
| Ventilator |  |  |  |  |  |  |  |  |  |  | |
| Rest |  |  |  |  |  |  |  |  |  |  | |
| V’E [L/min] | 13.6 (11-15.6) | 13 (10.6-15) | 14.9 (12.9-16) | 14 (11.8-15.6) | 13 (10.4-15.1) | 12.7 (10.5-15) | 14 (12-16) | 15.3 (14.8-15.3) | 14.8 (10.9-16) | 13.1 (10.3-14.9) | |
| Oxygen saturation [%] | 97 (96-98) | 97 (96-99) | 97 (96-98) | 97 (96-99) | 97 (96-98) | 97 (96-98) | 97 (96-99) | 99 (98-100) | 97 (95-99) | 98 (96.8-99) | |
| Breathing frequency [breaths/min] | 17.8 (15-21) | 17 (14.8-21) | 18.3 (15.2-21.2) | 18 (15.4-21.1) | 17.3 (14.8-21) | 16.4 (14.6-19) | 19.3 (15.6-21.3) | 17.8 (17.3-23.7) | 20.2 (16.4-21.4) | 16.3 (14.1-21) | |
| AT |  |  |  |  |  |  |  |  |  |  | |
| V’E [L/min] | 31.4 (25.3-37.1) | 31.5 (24.5-39.1) | 31.8 (28.6-35) | 31 (26.4-35) | 32.1 (24.5-40.2) | 33.6 (25-40.2) | 31 (26-36) | 31.4 (30.8-33) | 31.2 (26.2-45) | 26.6 (24-33.8) | |
| Oxygen saturation [%] | 97 (95-98) | 97 (95-98) | 97 (95-99) | 97 (95-98.5) | 97 (95-98) | 97 (95-98) | 97 (95-98) | 99 (98-100) | 97 (94.2-98) | 98 (96-98.5) | |
| Breathing frequency [breaths/min] | 22.3 (19.8-25.3) | 22.3 (19.8-25) | 22 (19.4-26.5) | 23.8 (19.8-27.4) | 22 (19.3-24) | 21 (18-24) | 23.2 (20.3-26.5) | 23.2 (22.8-27.3) | 24 (21.9-26.9) | 21.2 (19-24.1) | |
| Peak |  |  |  |  |  |  |  |  |  |  | |
| V’E [L/min] | 73.4 (61.1-90) | 73.2 (61.7-89.9) | 76.8 (59.3-90.4) | 73.5 (58.4-89.1) | 73 (63-92.1) | 80.8 (65-93.5) | 70.7 (56.5-88.6) | 69.6 (63-79.2) | 69.5 (56.4-83.1) | 67 (59.5-87.2) | |
| Oxygen saturation [%] | 96 (94-98) | 96 (94-97) | 96 (93-98) | 96 (94-98) | 96 (94-97) | 96 (94-98) | 96 (94-97) | 99 (98-99) | 96 (94-98) | 96 (95.8-97.2) | |
| Breathing frequency [breaths/min] | 37.2 (32.3-42.2) | 37 (33-41.4) | 38.1 (32.2-44.8) | 38 (32.1-40.7) | 37 (33-44.8) | 37 (32.1-41.8) | 38.1 (35-42.7) | 39.9 (36.6-42.7) | 38 (35-40.9) | 35.2 (30.4-42.2) | |
| (MVV-V’E)/ MVV [%] | 36.3 (27.6-43.1) | 37.9 (29.8-44.3) | 34.1 (23.5-41.6) | 38.4 (30.6-42.6) | 36.1 (23.7-43.5) | 36.3 (27.6-42.4) | 36.8 (27.7-43.5) | 23.4 (22.8-39) | 39.2 (30-45.7) | 38.4 (30.3-47.6) | |
| Abbreviations: AT: Anaerobic Threshold, HR: Heart Rate, IQR: Interquartile Range, MVV: Maximum Voluntary Ventilation, RER: Respiratory Exchange Ratio, V’E: Minute Ventilation, V’O2 : Oxygen Consumption, PE: Pulmonary Embolism, CPET: Cardiopulmonary Exercise Testing, P(ET-E)CO2: Partial pressure of end-tidal carbon dioxide, P(c-ET)CO2: Arterial minus end-tidal partial pressure of carbon dioxide, V’E/V’CO2: Minute ventilation to carbon dioxide output ratio, MVV: Maximum Voluntary  Ventilation. | | | | | | | | | | | |

| Table S4. Parameter CPET data per subgroup | | | | | | | | | | | | | | | | |
| --- | --- | --- | --- | --- | --- | --- | --- | --- | --- | --- | --- | --- | --- | --- | --- | --- |
| Frequency (%) | | | Overall sub-acute CPET | Lobar or (sub)segmental PE | Central PE | No RVD at baseline PE | RVD at baseline PE | low risk PE | Intermediate-high risk PE | No abnormal troponin | Abnormal troponin | Male | Female | BMI >= 30 | BMI <30 | |
| Number of patients included | | | 100 | 61 | 35 | 65 | 33 | 42 | 5 | 20 | 34 | 48 | 52 | 40 | 60 | |
| Metabolic | | |  |  |  |  |  |  |  |  |  |  |  |  |  | |
| Peak VO2 <80% of predicted | | | 23 (23) | 9 (15) | 13 (37) | 14 (22) | 9 (27) | 5 (12) | 3 (60) | 3 (15) | 15 (44) | 13 (27) | 10 (19) | 12 (30) | 11 (18) | |
| VO2 at AT <40% of predicted at peak | | | 10 (10) | 5 (8.3) | 4 (11) | 5 (7.8) | 4 (12) | 3 (7.3) | 1 (20) | 2 (11) | 4 (12) | 8 (17) | 2 (3.9) | 2 (5.1) | 8 (13) | |
| Peak RER < 1.05 | | | 28 (28) | 14 (23) | 13 (37) | 18 (28) | 9 (27) | 9 (21) | 1 (20) | 6 (30) | 11 (32) | 10 (21) | 18 (35) | 16 (40) | 12 (20) | |
| Peak heart rate <85% of pred | | | 15 (15) | 6 (9.8) | 9 (26) | 9 (14) | 6 (18) | 6 (14) | 0 (0) | 3 (15) | 8 (24) | 9 (19) | 6 (12) | 9 (22) | 6 (10) | |
| Cardiovascular/ gas exchange | | |  |  |  |  |  |  |  |  |  |  |  |  |  | |
| ΔVO2/Δload ≤ 8.4 mL/min/watt | | | 10 (10) | 6 (9.8) | 3 (8.6) | 6 (9.2) | 4 (12) | 5 (12) | 0 (0) | 3 (15) | 4 (12) | 4 (8.3) | 6 (12) | 4 (10) | 6 (10) | |
| Peak spO2 <90% or >5% drop during exercise | | | 4 (4) | 2 (3.3) | 2 (5.7) | 2 (3.1) | 2 (6.1) | 0 (0) | 0 (0) | 1 (5) | 2 (5.9) | 1 (2.1) | 3 (5.8) | 2 (5) | 2 (3.3) | |
| VE/VCO2 at AT ≥eqCO2 >= 34 | | | 28 (28) | 16 (27) | 10 (29) | 16 (25) | 11 (33) | 7 (17) | 4 (80) | 4 (21) | 13 (38) | 12 (25) | 16 (31) | 11 (28) | 17 (28) | |
| Peak O2 pulse <80% of pred | | | 27 (27) | 11 (18) | 15 (43) | 17 (26) | 10 (30) | 9 (21) | 4 (80) | 7 (35) | 15 (44) | 15 (31) | 12 (23) | 15 (38) | 12 (20) | |
| O₂-pulseAT/O₂-pulserest < 2.2 | | | 46 (46) | 24 (40) | 19 (54) | 28 (44) | 17 (52) | 16 (39) | 4 (80) | 9 (47) | 17 (50) | 19 (40) | 27 (53) | 21 (54) | 25 (42) | |
| O₂-pulseAT/O₂-pulserest ≥2.2 and 2.6 | | | 27 (27) | 17 (28) | 9 (26) | 15 (23) | 11 (33) | 8 (20) | 1 (20) | 6 (32) | 12 (35) | 13 (27) | 14 (27) | 12 (31) | 15 (25) | |
| Vd/Vt >30% | | | 27 (35) | 15 (33) | 10 (36) | 19 (36) | 7 (30) | 12 (32) | 0 (0) | 3 (17) | 9 (38) | 14 (37) | 13 (32) | 7 (23) | 20 (43) | |
| PETCO2 < 4.4 kPa (rest)or > 6.66 (peak) | | | 76 (76) | 43 (70) | 29 (83) | 46 (71) | 28 (85) | 27 (64) | 4 (80) | 16 (80) | 27 (79) | 32 (67) | 44 (85) | 32 (80) | 44 (73) | |
| P(ET-E)CO2 at max >1.7 kPa | | | 0 (0) | 0 (0) | 0 (0) | 0 (0) | 0 (0) | 0 (0) | 0 (0) | 0 (0) | 0 (0) | 0 (0) | 0 (0) | 0 (0) | 0 (0) | |
| P(c-ET)CO2 at max >0.3 kPa | | | 47 (56) | 25 (49) | 18 (62) | 33 (56) | 12 (52) | 21 (52) | 1 (25) | 10 (53) | 15 (58) | 23 (56) | 24 (56) | 18 (53) | 29 (58) | |
| Ventilator | | |  |  |  |  |  |  |  |  |  |  |  |  |  | |
| BR < 15% | | | 7 (7.2) | 5 (8.3) | 1 (2.9) | 3 (4.7) | 3 (9.4) | 3 (7.3) | 1 (20) | 1 (5) | 2 (5.9) | 2 (4.3) | 5 (10) | 4 (11) | 3 (5.1) | |
| Peak breathing frequency [breaths/min] ≥60 | | | 1 (1) | 1 (1.6) | 0 (0) | 0 (0) | 1 (3) | 0 (0) | 0 (0) | 0 (0) | 0 (0) | 1 (2.1) | 0 (0) | 0 (0) | 1 (1.7) | |
| Patterns | | |  |  |  |  |  |  |  |  |  |  |  |  |  | |
| Group A | 1. O2-pulse <80% of predicted OR O2-pulseAT/O2-pulserest <2. 6 | | 28 (36) | 17 (38) | 9 (32) | 16 (31) | 11 (48) | 10 (28) | 1 (25) | 8 (47) | 10 (42) | 13 (34) | 15 (38) | 14 (47) | 14 (30) | |
|  | 1. V’E/V’CO₂ at AT ≥34 OR Peak VD/VT >30% | | 8 (10) | 6 (13) | 2 (7.1) | 7 (13) | 1 (4.3) | 6 (17) | 0 (0) | 1 (5.9) | 0 (0) | 3 (7.9) | 5 (13) | 2 (6.7) | 6 (13) | |
|  | 1. O2-pulse<80% of predicted OR O2-pulseAT/O2-pulserest <2. 6 2. V’E/V’CO₂ at AT ≥34 OR Peak VD/VT >30% | | 30 (39) | 13 (29) | 15 (54) | 20 (38) | 9 (39) | 12 (33) | 3 (75) | 5 (29) | 13 (54) | 16 (42) | 14 (36) | 11 (37) | 19 (40) | |
| Group B | | 1. VO_2_ ≥80% of predicted 2. Peak O_2_-pulse ≥80% of predicted 3. O_2_-pulse_AT_/O_2_-pulse_rest_ ≥2.6 4. V’E/V’CO₂ at AT <34 5. Peak V_D_/V_T_ ≤30% | 11 (14) | 9 (20) | 2 (7.1) | 9 (17) | 2 (8.7) | 8 (22) | 0 (0) | 3 (18) | 1 (4.2) | 6 (16) | 5 (13) | 3 (10) | 8 (17) |  |
| Missing | | | 22 | 15 | 7 | 12 | 10 | 5 | 1 | 2 | 10 | 10 | 12 | 9 | 13 | |
| Abbreviations: AT: Anaerobic Threshold, HR: Heart Rate, IQR: Interquartile Range, MVV: Maximum Voluntary Ventilation, RER: Respiratory Exchange Ratio, V’E: Minute Ventilation, V’O2 : Oxygen Consumption, PE: Pulmonary Embolism, CPET: Cardiopulmonary Exercise Testing, P(ET-E)CO2: Partial pressure of end-tidal carbon dioxide, P(c-ET)CO2: Arterial minus end-tidal partial pressure of carbon dioxide, V’E/V'CO2: Minute ventilation to carbon dioxide output ratio, MVV: Maximum Voluntary Ventilation. | | | | | | | | | | | | | | | | |

| Table S5: OR of intermediate vs low risk PE | | | | | |
| --- | --- | --- | --- | --- | --- |
|  | peak V'O2 <80% pred | O2-pulse <80% pred | O_2_-pulse_AT_/O_2_-pulse_rest_ < 2.6 | V’E/V'CO2 >=35 | V_D_/V_T_ >30% |
| Intermediate risk PE vs low risk PE | 3.8 (1.2 to 14) | 1.9 (0.68 to 5.4) | NA | 2.7 (0.93 to 8.6) | 1 (0.35 to 3.1) |
| Abbreviations: V'O2: Volume of Oxygen Consumption, O2-pulse: Oxygen Pulse, V’E/V'CO2: Minute Ventilation to Carbon Dioxide Output Ratio, V_D_/V_T_: Dead Space to Tidal Volume Ratio | | | | | |

| Table S6. Overview CPET data in group B | |
| --- | --- |
| variable | CPET data |
| Number of patients included | 11 |
| Metabolic |  |
| Peak VO2 [mL/min] | 1930 (1790-2335) |
| Peak VO2 <80% of predicted | 0 (0) |
| VO2 at AT <40% of predicted at peak | 0 (0) |
| Peak load [watt] | 163 (141-202) |
| ΔVO2/Δload ≤ 8 mL/min/watt | 0 (0) |
| Cardiovascular/ gas exchange |  |
| VE/V'CO2 at AT | 27 (26-28.2) |
| VE/VCO2 at AT ≥34 | 0 (0) |
| Peak HR [beats/min] | 149 (134-156) |
| Peak heart rate <80% of pred | 0 (0) |
| Peak O2-pulse (VO2/HR) [mL/beat] | 13.9 (10.8-17.3) |
| Peak O2-pulse <80% of pred | 0 (0) |
| O₂-pulseAT/O₂-pulserest < 2.2 | 0 (0) |
| O₂-pulseAT/O₂-pulserest ≥2.2 and ≤2.6 | 0 (0) |
| Peak Vd/Vtalv [%] | 23 (13.8-28) |
| Vd/Vtalv >30% | 0 (0) |
| Peak P(c-ET)CO2 | 0.1 (-0.05-0.4) |
| P(c-ET)CO2 at max >0.5 kP | 4 (36) |
| Ventilator |  |
| Peak VE [L/min] | 71 (69.6-90.8) |
| Peak cxygen saturation [%] | 94 (93.5-96) |
| Peak spO2 <90% or >5% drop during exercise | 0 (0) |
| Peak breathing frequency [breaths/min] | 35 (30.7-41.9) |
| Peak breathing frequency [breaths/min] ≥60 | 0 (0) |
| Peak breathing reserve ((MVV-VE)/ MVV) [%] | 37 (35.1-45.2) |
| BR < 15% | 1 (10) |
| Abbreviations: AT: Anaerobic Threshold, HR: Heart Rate, IQR: Interquartile Range, MVV: Maximum Voluntary Ventilation, RER: Respiratory Exchange Ratio, V’E: Minute Ventilation, V’O2 : Oxygen Consumption, PE: Pulmonary Embolism, CPET: Cardiopulmonary Exercise Testing, P(ET-E)CO2: Partial pressure of end-tidal carbon dioxide, P(c-ET)CO2: Arterial minus end-tidal partial pressure of carbon dioxide, V’E/V’CO2: Minute ventilation to carbon dioxide output ratio, MVV: Maximum Voluntary  Ventilation. | |
